# Walnut Oil Alleviates Intestinal Inflammation and Restores Intestinal Barrier Function in Mice

**DOI:** 10.3390/nu12051302

**Published:** 2020-05-02

**Authors:** Adrian Bartoszek, Adam Makaro, Agnieszka Bartoszek, Radzisław Kordek, Jakub Fichna, Maciej Salaga

**Affiliations:** 1 Department of Biochemistry, Faculty of Medicine, Medical University of Lodz, 92-215 Lodz, Poland; adrian.bartoszek@stud.umed.lodz.pl (A.B.); adam.makaro@stud.umed.lodz.pl (A.M.); jakub.fichna@umed.lodz.pl (J.F.); 2 Department of Family Medicine and Community Nursing, Medical University of Lublin, 20-081 Lublin, Poland; agnieszka.bartoszek@umlub.pl; 3 Department of Pathology, Faculty of Medicine, Medical University of Lodz, 92-213 Lodz, Poland; radzislaw.kordek@umed.lodz.pl

**Keywords:** inflammatory bowel disease, ion transport, colon permeability, tight junction, free fatty acid receptors

## Abstract

Ulcerative colitis belongs to inflammatory bowel diseases, which is a group of chronic disorders of the gastrointestinal tract. It is a debilitating condition with a wide range of symptoms including rectal bleeding, diarrhea, and visceral pain. Current dietary habits often lead to imbalance in n-6/n-3 polyunsaturated fatty acids (PUFA) in favor of n-6 PUFA. Recent data showed the potential anti-inflammatory advantage of n-3 PUFA. Walnut oil (WO) is rich in those fatty acids and mainly consists of linoleic and linolenic acids that may act via free fatty acids receptors (FFARs). We assessed the anti-inflammatory effect of WO in the mouse model of dextran sulfate sodium (DSS)-induced colitis. Moreover, we examined changes in the expression of tight junction proteins (TJ), pro-inflammatory cytokines, and FFAR proteins in the inflamed mouse colon. WO improves the damage score in inflamed tissue, significantly restoring ion transport and colonic wall permeability. Inflammation caused changes in TJ, FFAR, and pro-inflammatory gene proteins expression, which WO was able to partially reverse. WO has anti-inflammatory properties; however, its exact mechanism of action remains unclear. This stems from the pleiotropic effects of n-3 PUFA ligands associated with receptor distribution and targeted signaling pathways.

## 1. Introduction

Ulcerative colitis (UC) belongs to the group of inflammatory bowel diseases, which are chronic disorders of the gastrointestinal (GI) tract. It is a heterogeneous disorder with contributions from genetic background, microbiota, and several environmental factors. It is not a fatal disease, but it is very debilitating with a wide range of symptoms such as rectal bleeding, vomiting, diarrhea, abdominal pain, anemia, and weight loss. Consequently, the chronicity of symptoms leads to a decrease in the patient’s quality of life [1]. Unfortunately, for now, there is no definitive cure for UC with only symptomatic treatments such as non-steroid anti-inflammatory drugs, steroids, immunomodulators, or biological agents [2].

The prevalence of UC is currently the highest in Europe (505 per 100,000 persons) followed by North America (286 per 100,000 persons), but in the last few decades, its incidence has been increasing in many countries worldwide, with a growing prevalence among children [3]. The disease may appear at any age with the first peak between 19 and 25 years old and the second peak in later life (>50 years of age) [4].

Within the last few decades, our diet has changed, leaving complex carbohydrates and fiber in favor of simple, processed carbohydrates; in parallel to that, the prevalence of IBD cases has risen [5]. Moreover, current dietary habits lead to imbalance in n-6/n-3 polyunsaturated fatty acids (PUFAs) in favor of n-6 PUFAs, especially in the Western diet [6]. Experimental evidence showed the potential advantage of n-3 PUFAs in IBD, particularly in UC where a high intake of n-3 PUFAs may lower the incidence of the disease. Unfortunately, the low number and poor quality of current studies made it impossible to imply guidelines on the use of n-3 PUFAs for treating UC patients [7].

Free fatty acids (FFAs), which have a non-esterified or unbound structure, are physiological ligands for specific G protein-coupled receptors (GPCRs), called Free Fatty Acid Receptors (FFARs). The family consists of four receptors, FFAR1–4, and each reacts to different aliphatic chain length FAs [8]. Medium-chain fatty acids (MCFAs) and long-chain fatty acids (LCFAs) with 7–12 and over 12 carbon atoms (CA), respectively, activate FFAR1 and FFAR4 [9]. FFAR2 and FFAR3 are stimulated by short-chain fatty acids (SCFAs), comprising 1–6 CA [10]. As FFARs joined the scientific stage not so long ago, only a few studies investigated their function in colitis [11]. FFAR1 stimulation was reported to ameliorate DSS-induced colitis [12]. On the other hand, research data for FFAR2 are contradictory, as some reports show that the activation of this receptor suppresses inflammation in mice [13,14], and the others demonstrate the opposite outcome [15,16]. FFAR3 has not been broadly studied in the context of IBD, with studies suggesting rather its pro-inflammatory action in animals [16]. Moreover, some studies showed the advantage of n-3 PUFA in animal models of colitis mediated through FFAR4 [17].

Walnut oil (WO) is rich in PUFAs, mainly linoleic (55%–70%) and α-linolenic (10%–18%) acids, and it is scanty in monounsaturated fatty acids (MUFAs) and saturated fatty acids (SFAs) [18]. Comparing to sunflower oil (SO), which is used as a reference in many studies due to its common usage in our diet, WO has a similar amount of MUFAs and n-6 PUFAs, but it has much more n-3 PUFAs [18].

The aim of this study was to characterize the anti-inflammatory action of a diet enriched in WO in the mouse model of colitis. We also investigated the effect of the diet on permeability and ion transport in an isolated mouse colon with the subsequent assessment of tight junctions (TJs), FFARs, and pro-inflammatory cytokines gene expression in mice fed with a standard diet (SD) or a diet enriched in SO or WO.

## 2. Materials and Methods

### 2.1. Animals and Study Design

**Figure 1:**
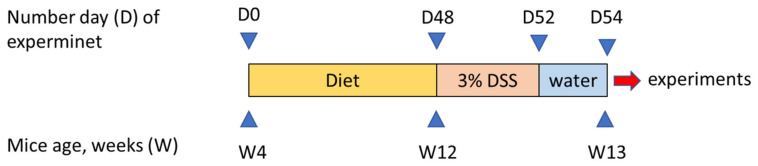
Scheme showing the experimental design of this study.

Naive male C57BL/6 mice obtained from the vivarium of the Mossakowski Medical Research Center Polish Academy of Sciences in Warsaw, Poland were used in all experiments. Animals weighed 12–18 g (4 weeks of age) at the beginning of experiment and were housed at a constant temperature (22 °C) and maintained under a 12-h light/dark cycle (lights on at 06:00) in sawdust-lined plastic cages. Tap water was provided ad libitum. Mice were randomly assigned to three groups fed with either SD containing 7% fat by weight, diet containing 7% SO, or diet containing 7% WO by weight. The content of FAs reflected the average amount of FAs ingested in humans. Animals were fed for 8 weeks (Figure 1). Body weight (BW) was measured every 4 days. All diets were formulated according to guidelines of the American Institute of Nutrition (AIN-93G) [19] to meet the nutritional requirements of growing mice and manufactured by the company specialized in animal food supply (ZooLab, Sędziszów, Poland). The content of FAs in the diets used in the study are listed in Table 1. The detailed composition of diets used in the study is listed in Appendix A.

All animal protocols were approved by the Medical University of Lodz Animal Care Committee (Protocol 21/ŁB134/2019) and complied with the European Communities Council Directive of 22 September 2010 of the EU (2010/63/EU). All efforts were made to minimize animal suffering and to reduce the number of animals used. Groups of 8 animals were used in all in vivo experiments.

### 2.2. Induction of Colitis

Dextran sulfate sodium (DSS; 3% wt/vol; molecular weight 40,000 (MP Biomedicals, Aurora, OH, Lot No. 5237K) was added to drinking water from day 0 of the experiment. On day 5, DSS solution was replaced with tap water, which was available for animals until the end of experiment (on day 7). On day 7, all mice were sacrificed, and the macroscopic score was performed. Briefly, the colon was isolated, opened longitudinally, and washed. The following parameters were assessed: macroscopic damage score (0–3), stool score (0–3), colon weight loss score (0–4), colon length loss score (0–4), and fecal blood (0–1). The colon tissue samples were also collected for analysis of myeloperoxidase (MPO) activity, as described previously [20]. All samples were collected in the same order starting from the distal part of the colon (MPO activity assessment, microscopic evaluation, mRNA isolation) from every single mouse. Control animals were receiving tap water throughout the whole experiment. Animal BW as well as general health and disease progression were monitored daily.

### 2.3. Determination of MPO Activity

One-centimeter segments of colon were weighed and homogenized in hexadecyltrimethylammonium bromide (HTAB) buffer immediately after isolation. Homogenates were centrifuged (13,200× *g*, 15 min), and 7 μL of supernatants were added to each well on a 96-well plate, containing 200 μL of 500 mM potassium phosphate buffer, supplemented with 0.167 mg/mL of O-dianisidine hydrochloride and 0.05 μL of 1% H_2_O_2_. Absorbance was measured at 450 nm. All measurements were performed in triplicate. MPO was expressed in milliunits per gram of wet tissue, 1 unit being the quantity of enzyme able to convert 1 μmol of H_2_O_2_ to water in 1 min at room temperature. Units of MPO activity per 1 min were calculated from a standard curve using purified peroxidase enzyme.

### 2.4. Histopathological Evaluation

After the macroscopic damage evaluation, segments of the distal colon were stapled flat, mucosal side up, onto cardboard and fixed in 10% neutral-buffered formalin for 24 h at 4 °C. Then, samples were dehydrated in sucrose, embedded in paraffin, sectioned at 5 μm, and mounted onto slides. Subsequently, sections were stained with hematoxylin and eosin and examined using an Axio Imager A2 microscope (Carl Zeiss, Oberkochen, Germany). Photographs were taken using a digital imaging system consisting of a digital camera (Axiocam 506 clolor, Carl Zeiss, Germany) and image analysis software (Zen 2.5 blue edition, Carl Zeiss, Germany). A microscopic total damage score was determined in a blind fashion based on the presence (score = 1) or absence (score = 0) of goblet cell depletion, the presence (score = 1) or absence (score = 0) of crypt abscesses, the destruction of mucosal architecture (normal = 1, moderate = 2, extensive = 3), the extent of muscle thickening (normal = 1, moderate = 2, extensive = 3), and the presence and degree of cellular infiltration (normal = 1, moderate = 2, transmural = 3) [21].

### 2.5. Ex Vivo Measurement of Epithelial Ion Transport

Epithelial ion transport was assessed according to the method described earlier [22]. Mice were sacrificed by cervical dislocation. Subsequently, full-wall thickness segments (0.5–1 cm) of the distal colon were isolated and immediately placed in an Ussing chamber (Physiologic Instruments, Inc., San Diego, CA, USA) containing 6 mL of Krebs solution of the following composition (mM): NaCl, 115; KH_2_ PO_4_, 2; MgCl_2_, 2.4; NaHCO_3_, 25; KCl, 8; CaCl_2_, 1.3. Krebs solution was saturated with 95% O_2_ and 5% CO_2_ and contained glucose (10 mM) and mannitol (10 mM) added to the basolateral and mucosal side, respectively. The bath temperature was maintained at 37 °C. The exposed surface of the tissue was 0.3 cm^2^. Tissues were voltage clamped to zero, using the WPI EVC-4000 voltage clamp apparatus (World Precision Instruments, Sarasota, FL, USA) with an Ag/AgCl electrode and 3 M KCl agar bridge. Once a stable baseline in a short circuit current (Isc, mA/cm^2^) was achieved (15–30 min), the tested FFAR agonist GW9508 (10^–5^ M, FFAR1 agonist), 4-CMTB (10^–5^ M, FFAR2 agonist), AR 420626 (10^–5^ M, FFAR3 agonist), and GSK 137647 (10^−5^ M, FFAR4 agonist), dissolved in DMSO or an equal volume of vehicle (DMSO, final concentration: 0.1%) was added to the basolateral side of the chamber. Ten minutes later, preparations were challenged with either forskolin (FSK) (cAMP-dependent secretagogue, 10^–5^ M) or veratridine (VER) (voltage-dependent Na^+^ channel activator, 3 × 10^–5^ M) [20]. For each challenge, the peak change in Isc (∆Isc) was determined.

### 2.6. Ex Vivo Assessment of Intestinal Permeability

For measurements of intestinal permeability, full-wall thickness segments of the mouse colon were opened along the mesenteric border and mounted in Ussing chambers (0.3 cm^2^ opening), exposing mucosal and serosal surfaces to 6 mL of oxygenated Krebs buffer. Mannitol and glucose (both 10 mmol) were added to the mucosal and serosal side, respectively, and the tissues were allowed to equilibrate for 15 min. The experiment was initiated by replacing the buffer in the mucosal chamber with 10 mL of Krebs buffer containing fluorescein isothiocyanate dextran (FITC–dextran; Sigma-Aldrich, St. Louis, MS, USA) at a concentration of 1 mg m/L. Then, chambers were covered to prevent light exposure. Fluorescence intensity in the serosal chamber was measured immediately to determine baseline fluorescence. Samples were taken from the serosal chambers at 20-min intervals for a total period of 120 min, and FITC–dextran concentration was measured at an excitation wavelength of 494 nm and emission wavelength of 521 nm.

### 2.7. RNA Isolation, Reverse Transcription and qPCR

Briefly, RNA was isolated from the distal sections of colon (weighing 20–30 mg) obtained from healthy and DSS-treated animals, according to manufacturer’s protocol using a Total RNA Mini Plus kit (A&A Biotechnology, Gdansk, Poland). RNA was eluted from ion exchange columns by diethyl pyrocarbonate (DEPC)-treated water (40 μL). Then, we purified total RNA, using Lithium Chloride Precipitation Solution according to the manufacturer’s protocol (Life Technologies, Carlsbad, CA, USA). The purity and quantity of isolated RNA was measured using Colibri Microvolume Spectrophotometer (Biocompare, San Francisco, CA, USA). Total RNA (2 μg) was transcribed onto cDNA with a high capacity Reverse Transcriptase Kit (Life Technologies, Carlsbad, CA, USA) according to the manufacturer’s protocol. Quantitative analysis of the expression was performed using fluorescently labeled probes (Life Technologies, Carlsbad, CA, USA): OCLN (Mm00500912_m1), CLDN1 (Mm00516701_m1), CLDN2 (Mm00516703_s1), CLDN3 (Mm00515499_s1), CLDN4 (Mm00515514_s1), CLDN7 (Mm00516817_m1), CLDN10 (Mm01226326_g1), CLDN12 (Mm01316510_m1); FFAR1 (Mm00809442_s1), FFAR2 (Mm02620654_s1), FFAR3 (Mm02621638_s1), FFAR4 (Mm00725193_m1), TNF-α (Mm00443258_m1), interleukin 1 β (Il-1β) (Mm00434228_m1), and glyceraldehyde-3-phosphate dehydrogenase (GAPDH) (Mm99999915_g1) as endogenous control on a Mastercycler S realplex 4 apparatus (Eppendorf, Hamburg, Germany) using TaqMan Gene Expression Master Mix (Life Technologies, Carlsbad, CA, USA) in accordance with the manufacturer’s protocol. All experiments were performed in triplicate. The Ct (threshold cycle) values for studied genes were normalized to Ct values obtained for GADPH. The relative amount of mRNA copies was calculated using the following equation: 2^-ΔCt^×1000.

### 2.8. Reagents and Drugs

All drugs and reagents, unless otherwise stated, were purchased from Sigma-Aldrich (Poznan, Poland). DSS (MW 40,000) was purchased from MPBiomedicals (Aurora, OH, USA). FFAR1–4 ligands were purchased from Bio-Techne (Warsaw, Poland).

### 2.9. Statistics

In *in vivo,* ex vivo, and in vitro experiments, n indicates the number of animals used in the experiment (one sample per animal was obtained for each MPO activity measurement, qPCR, hematoxylin and eosin (H&E) stain, FITC experiment, and ΔIsc measurement). Statistical analyses were performed using Prism 8.0 (GraphPad Software Inc., La Jolla, CA, USA). The data are expressed as means ± SEM.

For the analysis of the effects on ion transport, colon permeability, and expression of TJs, FFARs, and pro-inflammatory cytokines, a one-way ANOVA was performed followed by Newman–Keuls post hoc tests. Ordinal data were analyzed by Kruskal–Wallis one-way analysis of variance. Values of *p* < 0.05 were considered statistically significant.

## 3. Results

### 3.1. WO Diet Does Not Cause Excessive Body Weight Gain in Mice and Alleviates Symptoms of DSS-Induced Colitis

**Figure 2:**
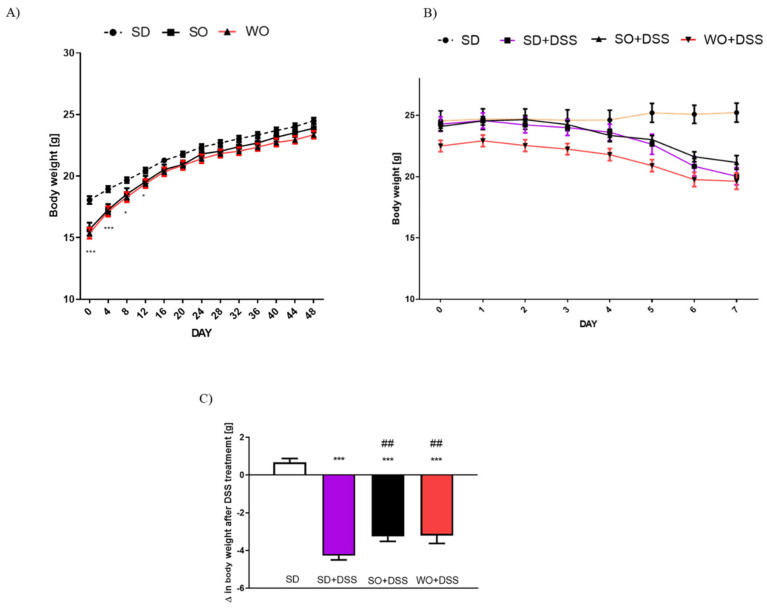
Data for body weight changes (**A**) in mice fed with standard diet (SD), SD supplemented with sunflower oil (SO), and SD supplemented with walnut oil (WO). Data for body weight loss in the course of dextran sulfate sodium (DSS) treatment (**B**) and overall change in body weight after 7 days of colitis (**C**). Data represent mean ± SEM, n = 28 per group (**A**); n = 8 per group (**B**,**C**); * *p* < 0.05, ** *p* < 0.01, *** *p* < 0.001, as compared to SD; ## *p* < 0.05, as compared to SD + DSS.

Mice at 4 weeks of age were randomly assigned to three groups fed with either SD, SO, or WO diet, and their BW was recorded for 8 weeks (measurement every 4 days) (Figure 2A). Since day 16, there was no significant difference between groups (Figure 2A).

**Figure 3:**
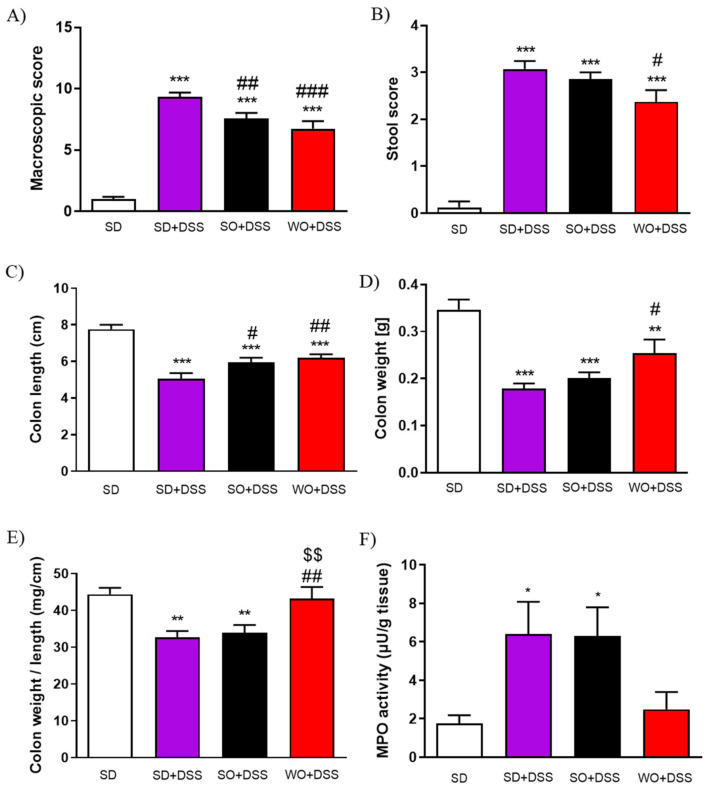
The anti-inflammatory effect of sunflower oil (SO) and walnut oil (WO) in a mouse model of colitis induced by DSS. Figure shows the data for macroscopic score (**A**), stool score (**B**), colon length (**C**), colon weight (**D**), colon weight/colon length ratio (**E**), and myeloperoxidase (MPO) activity (**F**). Data represent mean ± SEM, n = 8. * *p* < 0.05, ** *p* < 0.01, *** *p* < 0.001, as compared to control; # *p* < 0.05, ## *p* < 0.01, ### *p* < 0.001 as compared to DSS; $$ *p* < 0.01, as compared to sunflower oil (SO).

On day 48 of the experiment, the tap water was changed into the 3% DSS solution. After 7 days of colitis, the largest BW loss was observed in SD + DSS group (Figure 2A,B). Treatment with SO and WO significantly reduced BW loss (Figure 2B). On day 7, mice were sacrificed, the macroscopic score was performed, and the tissue for both histological assay and rt-PCR analysis were collected. We found that DSS treatment significantly increased the macroscopic score, stool score, and lowered the colon length and colon weight in all groups (Figure 3A–D). The WO-enriched diet was able to alleviate the effect of DSS, while SO only increased the colon length. Interestingly, the colon weight/length ratio was restored in the WO group completely, as there was no difference compared to the control group (Figure 3E). MPO activity was increased in both SD + DSS and SO + DSS groups comparing to controls, while WO + DSS did not differ significantly from non-inflamed mice (Figure 3F).

**Figure 4:**
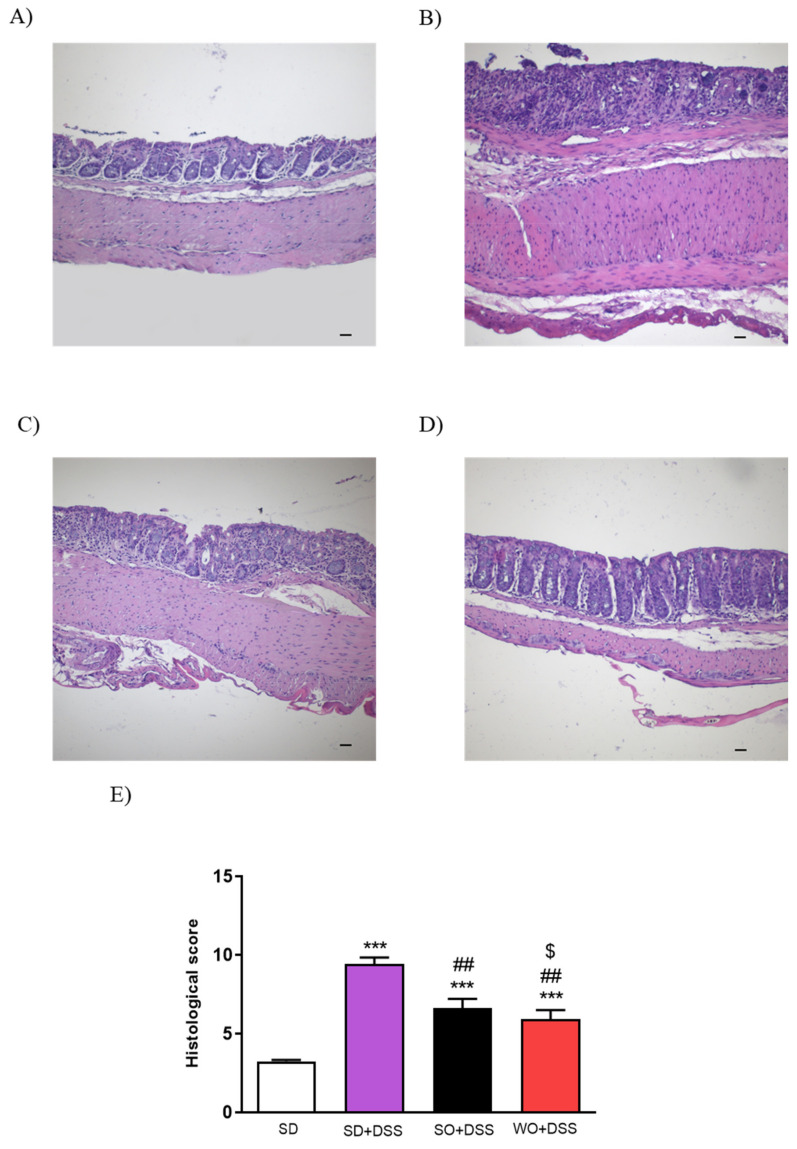
Representative micrographs of hematoxylin and eosin-stained sections of distal colon from (**A**) SD, (**B**) SD + DSS, (**C**) SO + DSS, (**D**) WO + DSS, and microscopic total damage score (**E**). Scale bar = 100 μm. Data represent mean ± SEM, n = 8. *** *p* < 0.001, as compared to control; ## *p* < 0.01, ### *p* < 0.001 as compared to DSS; $ *p* < 0.05, as compared to sunflower oil (SO).

Histological assessment of mouse colon samples supported the macroscopic observations (Figure 4). Analysis revealed intact epithelium, normal muscle architecture, and an absence of edema in the control group (Figure 4A). Microscopic damage, characterized by the loss of mucosal architecture, the presence of crypt abscesses, the thickening of smooth muscle, as well as widespread cellular infiltration was observed in SD + DSS as well as SO + DSS groups (Figure 4B,C). WO prevented colon damage, as examined microscopically (Figure 4D) and in the histological score (Figure 4E).

### 3.2. WO Inhibits the Effect of DSS on Epithelial Ion Transport in the Mouse Colon

**Figure 5:**
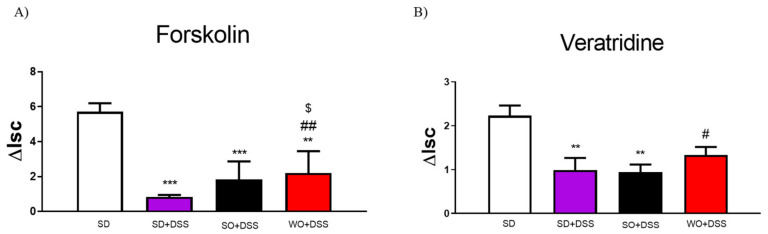
Changes in forskolin-stimulated (**A**) and veratridine-stimulated (**B**) short-circuit current (ΔIsc) in the colon of mice fed with diet enriched with sunflower oil (SO) and walnut oil (WO) and treated with DSS. Data represent mean ± SEM, n = 8. ** *p* < 0.01, *** *p* < 0.001, as compared to control; # *p* < 0.05, ## *p* < 0.01 as compared to DSS; $ *p* < 0.05, as compared to sunflower oil (SO + DSS).

We also investigated the effect of SO and WO oils on epithelial ion transport in isolated mouse colon, which were mounted in Ussing chambers. To investigate the cyclic adenosine monophosphate (cAMP) -dependent mechanism, we used FSK. ΔIsc values were reduced in inflamed tissues as compared to controls in all colon samples challenged with FSK (Figure 5A). Treatment with WO significantly increased cAMP-dependent transport as compared to both SD + DSS and SO + DSS groups (Figure 5A).

To examine ion flow contingent on the voltage-dependent Na^+^ channel, we used VER. DSS significantly lowered the ΔIsc in SD + DSS and SO + DSS groups (Figure 5B). WO reversed this effect in inflamed tissue.

### 3.3. WO Inhibits the Effect of DSS on Colon Permeability Ex Vivo

**Figure 6:**
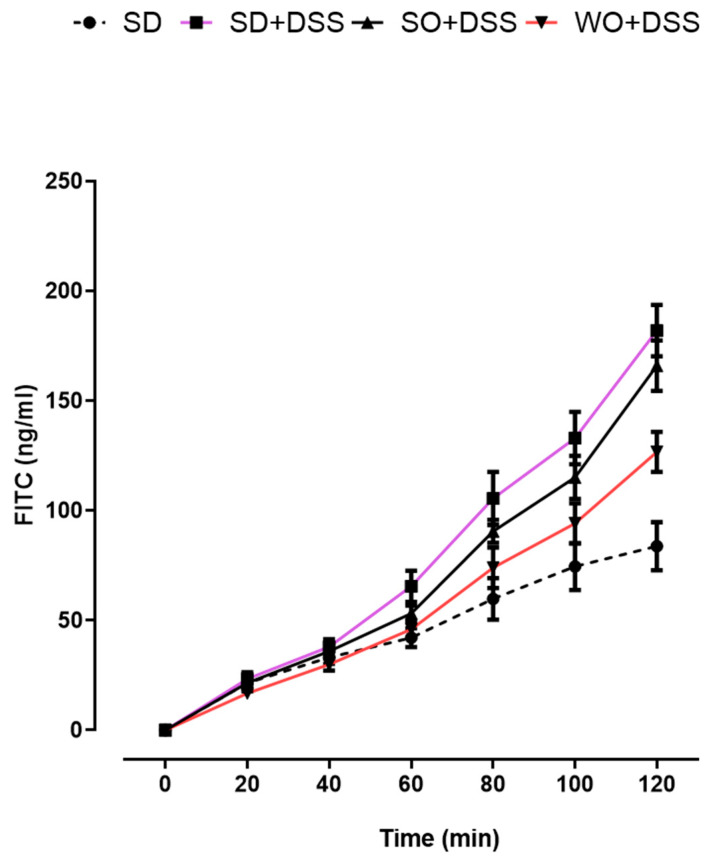
Effect of diet enriched with sunflower oil (SO) and walnut oil (WO) on colon permeability in DSS-treated mice. Data represent mean ± SEM, n = 8 animals per group. SD and SD + DSS groups are significantly different starting from 80 min (80 min, *p* < 0.001; 100 min, *p* < 0.001; 120 min, *p* < 0.001). SD + DSS and WO + DSS groups are significantly different starting from 80 min (80 min, *p* < 0.05; 100 min, *p* < 0.01; 120 min, *p* < 0.001).

To evaluate the paracellular permeability in mouse colon segments, the mucosal-to-serosal macromolecular flux was measured using a fluorescent probe. As shown in Figure 6, permeability to FITC-labeled dextran was increased in the colon of DSS-treated mice at 80, 100, and 120 min after the addition of the probe to the mucosal chamber, comparing to controls. We did not observe any differences caused by the SO-enriched diet. On the other hand, we found that in the colon segments of mice fed with a WO-enriched diet, the effect of DSS on paracellular permeability was inhibited at 80, 100, and 120 min after the addition of the FITC dextran probe. At 120 min, SO + DSS and WO + DSS groups were significantly different (*p* < 0.01).

### 3.4. Regulation of TJs, FFARs, and Pro-Inflammatory Cytokines Gene Expression by the WO in the Mouse Colon

**Figure 7:**
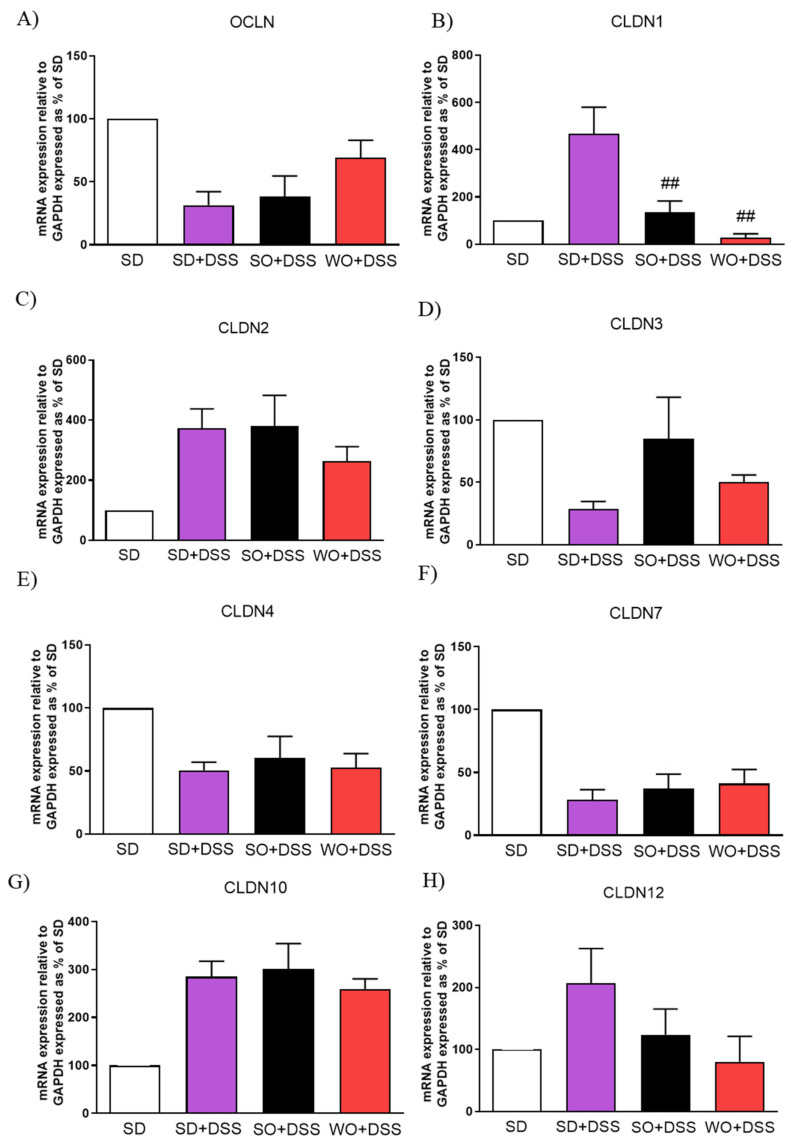
Changes in tight junction genes expression (**A–H**) in the colon of mice treated with DSS and fed with standard diet (SD), sunflower oil (SO), and walnut oil (WO). Values expressed as percent of control group. Data represent mean ± SEM, n = 8–12. ## *p* < 0.01, as compared to DSS.

To assess TJs mRNA expression, the real-time PCR was performed using distal colon samples collected from every group (Figure 7). Both SO and WO were able to significantly restore the level of CLDN1 (Figure 7B). We did not observe any changes in CLDN12 in investigated groups (Figure 7H).

**Figure 8:**
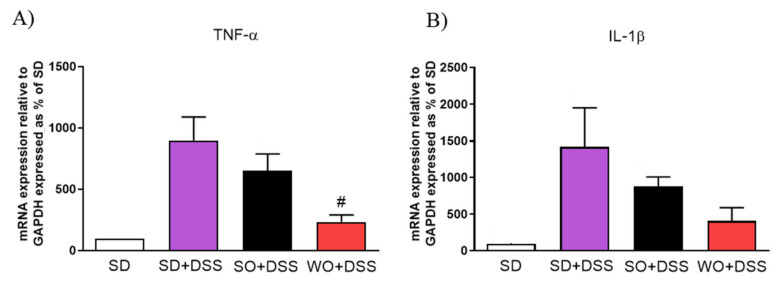
Changes in pro-inflammatory cytokines gene expression (**A**,**B**) in the colon of mice treated with DSS and fed with standard diet (SD), sunflower oil (SO), and walnut oil (WO). Values expressed as percent of control group. Data represent mean ± SEM, n = 8–12. # *p* < 0.05, as compared to DSS.

Supplementation with WO significantly inhibited the effect of DSS on tumor necrosis factor α (TNF-α) (Figure 8A) and partially on interleukin 1β (Figure 8B).

**Figure 9:**
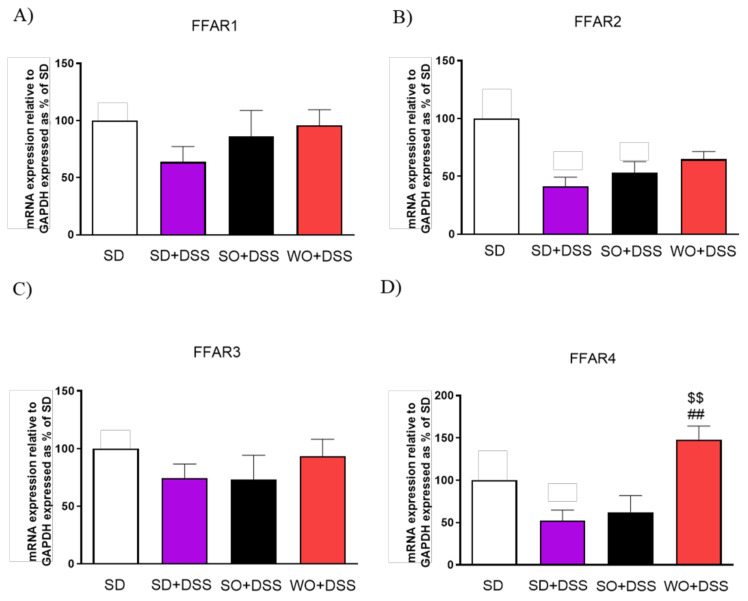
Changes in free fatty acid receptors genes expression (**A–D**) in the colon of mice treated with DSS and fed with standard diet (SD), sunflower oil (SO), and walnut oil (WO). Values expressed as percent of control group. Data represent mean ± SEM, n = 8–12. ## *p* < 0.01, as compared to DSS, $$ *p* < 0.01, as compared to sunflower oil (SO).

Diet enriched in FAs did not alter FFAR1 and FFAR3 expression (Figure 9A,C). Mice fed with a WO-enriched diet exhibited significantly higher FFAR4 levels than the SD + DSS group (Figure 9D).

## 4. Discussion

Ulcerative colitis is still incurable, and its effective treatment is difficult to achieve. Most currently used therapies cause only temporary remission and induce many adverse effects. For active disease, 5-aminosalicylic acid (5-ASA) is the preferred treatment, with topical use being more effective than oral administration [23]. If the patient does not respond, the addition of oral corticosteroids or biologic drugs may be effective [24]. In many cases, pharmacological protection from the disease relapse fails, and then the surgical intervention is necessary [25]. To avoid the surgery, many patients reach for a different therapeutic option: immunotherapy, which is not effective in inducing remission of active UC; however, it may prevent the relapse, as revealed in a recent meta-analysis [26].

The above-mentioned therapies are not well-tolerated and cause systemic side effects, which are especially problematic, as the therapy is life-long. As there is no effective treatment and the rate of UC in the early age is increasing, there is an urgent need for new, safe, and well-tolerated therapeutics. Here, we propose a new strategy based on a diet enriched in WO in order to alleviate symptoms associated with colitis. To the best of our knowledge, this is the first study evaluating the effect of WO in IBD.

In this study, we did not observe any excessive BW resulting from a diet, which is a desirable result, since we can exclude obesity-related effects. [27]. On the other hand, in the course of colitis, animals’ BW decreased significantly comparing to controls, which is what we expected. Diets enriched in oils significantly inhibited the BW loss after 7 days of DSS treatment, which is in line with a study by Zhao et al. demonstrating a similar effect caused by docosahexaenoic acid in the model of colitis induced by interleukin-10 deficiency [28]. The primary end point of our study was the macroscopic assessment of the colon changes. In mice receiving SD + DSS, colon weight and length were significantly lowered in comparison to controls. WO partially reversed the effect of DSS on all measured parameters, which corresponds with BW change. Of note, SO also improved some of the assessed parameters, although its anti-inflammatory activity was much lower than WO. We also found that the index colon weight/colon length was significantly improved compared to the DSS group. The microscopic evaluation of colon sections corresponds with macroscopic results. In line with our observations, many previous studies involving both mice and humans [17,29,30] showed the anti-inflammatory effect of omega fatty acids in the GI tract. Vilaseca et al. compared the influence of a cod liver oil (n-3) and sunflower oil (n-6)-enriched diet in 2,4,6-trinitrobenzenesulfonic acid solution (TNBS)-induced colitis in rats [31]. Animals supplemented with n-3 FAs exhibited a markedly reduced inflammatory response, comparing to the n-6 diet, and those sacrificed 20 days later than the first group had almost no inflammation [31]. Comparison of the anti-inflammatory effect of different FAs in TNBS-induced colitis demonstrated the following efficacies: α-linolenic acid > linolenic acid [32]; fish oil > cotton and sunflower oils [33]; perilla oil > safflower oil [34]; fish oil > soybean and coconut oils, pig brain phospholipids [35]. In mice lacking the IL-10 gene and exposed to DSS, the inflammation was reduced in the fish oil group compared to the corn oil group [36]. Moreover, transgenic mice expressing the fat-1 gene encoding n-3 FAs desaturase that produces n-3 FAs from n-6 acids were used to examine the impact of enhanced n-3 PUFA tissue status on the development of colitis. The macroscopic score was lower in fat-1 (supplemented with safflower oil) than in wild-type mice in the DSS model of colitis [37]. The same study showed a significant inhibition of nuclear factor κB (NF-κB) and decreased activity of cytokines mediated by this pathway: IL-1β and TNFα. [37]. Another study suggested an additional mechanism underlying anti-inflammatory activities of PUFAs. The n-3 PUFAs downregulate peroxisome proliferator-activated receptor γ pathway in intestinal Caco-2 cells [38]. Moreover, linolenic acid influences the immunological processes involved in colitis development. This fatty acid disrupts the Th1/Th2/Th17 pathways involved in inflammatory bowel disease (IBD) pathogenesis and activates the Treg pathway. It is proven that the predominant cytokine involved in the Treg pathway, Il-10, acts as an anti-inflammatory factor [39]. Another proposed mechanism assumed that the omega fatty acids, as precursors for eicosanoids, modulate the inflammatory response. In our study, we used WO, which is rich in PUFAs, mainly linoleic and linolenic acids. The former is metabolized to leukotrienes and prostanoids that are pro-inflammatory mediators, and the latter is metabolized to leukotrienes and eicosanoids, which have anti-inflammatory effects. Both linoleic and linolenic acid compete for the same enzymes in their metabolic routes, so the proportion of the acids is important for the final outcome [40]. In the Western diet, the typical ratio of n3/n6 fatty acids is estimated to be 0.058–0.067 [6], while the ratio in WO is approximately 0.25 [40]. In our study, the fatty acids balance is congruent with previous reports. WO not only has the best n3/n6 ratio but also the highest absolute n-3 content. On the other hand, SO may have a slightly better n3/n6 balance than SD, which could explain some of the anti-inflammatory effects in this group. Finally, diets differed only in the used oil, so we suggest that all observed effects derived from fatty acids. Secretory diarrhea, a major symptom of UC, may result from bacterial toxins and/or the disrupted flow of ions, including Na^+^ and Cl^-^ [41]. In UC, the immune system modulates the absorption of electrolytes by the release of cytokines and by the influence on the enteric nervous system [42]. Therefore, in this study, we aimed to elucidate the potential of WO to restore physiological ionic flow in the colon. We used FSK, which is naturally occurring diterpene that is useful in studying mechanisms underlying secretory diarrhea. It activates adenylate cyclase via direct action on the enzyme catalytic subunit and has been shown to affect cAMP-dependent cellular functions [43]. cAMP activates protein kinase A (PKA), causing an increase in Cl^–^ and water secretion into the lumen through cystic fibrosis transmembrane conductance regulator (CFTR) channels, and it restrains water absorption through the phosphorylation of Na^+^/H^+^ exchanger isoform 2/3 (NHE2/3) regulatory proteins [44]. Here, we examined the effect of SO and WO on FSK-induced epithelial ion transport. It was previously reported that in DSS colitis, the ion transport is suppressed [45]. Similarly, in our study, mice treated with DSS had reduced ion transport compared to the control group. SO lowered ∆Isc, while WO was able to partially reverse ∆Isc change.

We also tested the effect of FAs on VER-induced ion flow. VER is commonly used to investigate the effect of neural stimulation on the intestinal barrier. This compound depolarizes enteric neurons as consequence of elevated voltage-sensitive Na^+^ permeability, and it subsequently causes epithelial Cl^–^ secretion across the colonic mucosa [46]. Here, DSS also suppressed the VER-induced ∆Isc values. However, WO was able to reverse this effect. Following the results, we could assume that a diet enriched in WO might be a good direction in treating diarrhea or so-called leaky gut. Moreover, it was previously showed that a long-term diet enriched in oleic acid (one of the LCFAs) in a DSS model of colitis delayed diarrhea and rectal bleeding but did not improve colonic histopathology compared to controls [47].

Then, we decided to investigate the effect of the diet on colon permeability in the setting of colonic inflammation. The amount of FITC that passed through inflamed colon, mounted in a Ussing chamber, significantly differed from the control. We found that WO inhibited the effect of DSS on paracellular permeability in the mouse ileum at 80, 100, and 120 min after the addition of the FITC dextran probe. It complies with epithelial ion transport assessment and suggests its potential in the alleviation of UC symptoms caused by disruption of the intestinal epithelial barrier.

Recently, researchers’ attention has focused on the damage and abnormal functioning of the intestinal epithelial barrier occurring in IBD and the role of TJs [48]. TJ, which are the main constituent of gut epithelial barrier at the molecular level, are intercellular adhesion complexes, which maintain cell polarity by restricting the movement of proteins within the plasma membrane and regulating paracellular solute and water flux. TJs are composed of transmembrane proteins, including occludin (OCLN) and claudins (CLDN). Both loss-of-function and gain-of-function of TJs may lead to a change in selective ion permeability [48]. Mice overexpressing CLDN1 were more susceptible to colonic inflammation and demonstrated impaired recovery following the DSS treatment [49]. Likewise, the up-regulation of CLDN2 in the mouse gut showed its crucial role in intestinal homeostasis by regulating epithelial permeability, inflammation, and proliferation [50]. The knockout of CLDN7 in mice caused serious intestinal defects, including mucosal ulcerations and inflammation, which leads to death [51]. Interestingly, CLDN8 contributed to the regulation of paracellular Na^+^ permeability, protecting the leakage of Na^+^ into the intestinal lumen [52]. Western blot analysis revealed no difference in the expression of CLDN1 and CLDN2 in the distal colon of inflamed mice as compared with control group. However, OCLN expression was reduced in mice treated with DSS [53]. Another research group found that the expression of both OCLN and CLDN3 is reduced and that CLDN1 is not affected in DSS-induced colitis compared to the control group [54]. Here, we assessed TJs mRNA expression with the use of real-time PCR. Interestingly, studies among IBD patients revealed that the level of CLDN2 is increased [28], and the levels of CLDN3, CLDN4, CLDN7, and OCLN decreased [29], which complies with our outcomes in the animal model.

MCFAs and LCFAs are mainly derived from food, the lipolysis of adipose tissue, or biosynthesis [55]. These FAs activate FFARs. Their role in the treatment of inflammatory states such as type 2 diabetes, metabolic syndrome, cardiovascular diseases, and asthma is well-established [8,10]. As recent reports showed their role in colitis [11], we also decided to investigate whether the expression of FFARs is changed by our dietary interventions. We found that FFAR4 is significantly overexpressed in mice fed with WO comparing to SD. In line with our outcomes, Cheshmehkani et al. found that feeding rats for 8 weeks with an n-3-enriched diet (fish oil) up-regulated FFAR4 expression in the colon and inhibited TNF-α [56]. Of note, both FFAR1 and FFAR4 were shown to be overexpressed in CD patients and were positively correlated with TNF-α levels [57], which suggests that TNF-α induces FFAR4 expression. This partial contradiction with our results may stem from the fact that tissues were obtained from the ileal mucosa of CD patients, whereas we collected samples from the distal colon of mice exposed to DSS, which mimics UC.

One of the proposed mechanisms underlying possible FFARs involvement in anti-inflammatory response is the impact on β-arrestin-2. Glucagon-like peptide-1 (GLP-1) secretion was reported to suppress inflammation. It was shown that the stimulation of FFAR4 in STC-1 cells increased GLP-1 secretion [58]. The incubation of Caco-2 cells with selective agonists of FFAR1 (GW9508) and FFAR4 (TUG-891) [59] as well as the treatment of GLUTag mouse enteroendocrine cell lines with TUG-891 also elevated GLP-1 excretion [60]. On the other hand, supplementation with fish oil for eight weeks did not alter the GLP-1 level in rats [56]. However, it has to be considered that GLP-1 is rapidly inactivated by dipeptidyl peptidase-4 in vivo, and the changes in plasma levels might have not been detected in the weekly measurements [56]. The FFAR4 agonist TUG-891 showed anti-inflammatory properties by binding to β-arrestin-2 and the inhibition of nuclear factor κB (NF-κB) [59]. FFAR4 activation causes the recruitment and subsequent internalization of β-arrestin 2, which then forms a complex with TAK1-binding protein (TAB1), hence preventing TAK1—TAB1 association. In the absence of TAB1, TNF-α cannot activate TAK1, which results in blocking the downstream TNF-α inflammatory pathways [28].

## 5. Conclusions and Future Perspectives

This study is a step toward establishing novel dietary recommendations for patients suffering from IBD. WO improved symptoms of inflammation and sustained the intestinal barrier in the distal colon. In addition, IBD is a chronic, relapsing inflammation that elevates the risk of cancer progression. Thus, by treating the disease with WO, we could prevent patients from colitis-associated cancer.

One of the proposed mechanisms underlying the action of WO includes FFARs. Difficulties in the interpretation of the data, especially regarding FFARs, stem from the pleiotropic effects of FAs associated with receptor distribution and different signaling pathways. This warrants further studies to fully exploit the potential of WO in IBD.

## Figures and Tables

**Table 1:** Composition of fatty acids in diets used for experiments.

Fatty Acid Content (%)	Soybean Oil	Sunflower Oil	Walnut Oil
Palmitic acid C 16:0	7–10	2–9	6–8
Stearic acid C 18:0	2–5	2–7	1–3.5
Oleic acid C 18:1	22–30	75–91	14–21
Linoleic acid C 18:2	50–60	3–17	54–65
α-Linolenic acid C 18:3	2–5	Max 0,3	9–16
n-3/n-6 balance	0.04–0.083	0.001–0.1	0.167–0.246

## Appendix A

**Table A1:** Composition of diets that were included in the study.

Ingredients (g/kg Diet)	SD a	SD Supplemented with 7% Sunflower oil (SO)	SD Supplemented with 7% Walnut Oil (WO)
Corn starch	397.486	397.486	397.486
Casein	200.00	200.00	200.00
Maltodextrin	132.00	132.00	132.00
Sucrose	100.00	100.00	100.00
Soybean oil	70.00	0.00	0.00
Sunflower oil	0.00	70.00	0.00
Walnut oil	0.00	0.00	70.00
Fiber	50.00	50.00	50.00
aAIN93G Mineral mix	35.00	35.00	35.00
aAIN93G Vitamin mix	10.00	10.00	10.00
L-Cystine	3.00	3.00	3.00
Choline bitartrate	2.50	2.50	2.50
Tert-butylohydrochinon	0.014	0.014	0.014
	1000.0	1000.0	1000.0

Full ingredient list for the diets in this study, formulated by ZooLab, Poland. a: SD refers to rodent diet 93 of the American Institute of Nutrition.
